# Air pollution during New Year’s fireworks and daily mortality in the Netherlands

**DOI:** 10.1038/s41598-019-42080-6

**Published:** 2019-04-05

**Authors:** Frans E. Greven, Judith M. Vonk, Paul Fischer, Frans Duijm, Nienke M. Vink, Bert Brunekreef

**Affiliations:** 1 Department of environmental health, municipal health services Groningen, PO Box 584, 9700 AN Groningen, The Netherlands; 2 Department of epidemiology, university of Groningen, university medical center Groningen, PO Box 30.001, 9700 RB Groningen, The Netherlands; 3 Groningen research institute on asthma and COPD (GRIAC), university of Groningen, university medical center Groningen, PO Box 30.001, 9700 RB Groningen, The Netherlands; 4 Centre for Sustainability, Environment and Health; Department for Environmental Health, National Institute of Public Health and the Environment, RIVM, PO Box 1, 3720 BA Bilthoven, The Netherlands; 5 Institute for Risk Assessment Sciences, University of Utrecht, Yalelaan 2, 3584 CM Utrecht, The Netherlands; 6 Julius Center for Health Sciences and Primary Care, University Medical Center Utrecht, 3584 CJ Utrecht, The Netherlands

## Abstract

Short-term exposure to air pollution has been associated with cardiovascular and respiratory mortality and morbidity. Little is known about associations between air pollution caused by firework events and daily mortality. We investigated whether particulate matter from fireworks during New Year’s celebrations was associated with daily mortality. We analyzed the celebrations of the years 1995–2012. PM_10_ concentrations increased dramatically during the firework events. Countrywide, the daily average PM_10_ concentrations from 27–30 December was 29 μg/m^3^ and increased during the first hour of the New Year by 277 μg/m^3^. In the more densely populated areas of the Netherlands the increase was even steeper, 598 μg/m^3^ in the first hour of the New Year. No consistent associations were found using linear regression models between PM_10_ concentrations during the first six hours of 1 January and daily mortality in the general population. Yet, using a case-crossover analysis firework-days and PM_10_ concentrations were associated with daily mortality. Therefore, in light of the contradictory results obtained with the different statistical analyses, we recommend further epidemiological research on the health effects of exposure to firework emissions.

## Introduction

Setting off fireworks during events such as the Diwali in India^1–4^, Independence Day in the USA^5^, Lantern Festival in China and Taiwan^6,7^, Guy Fawkes in the UK^8,9^ and, in many countries, New Year’s celebrations^10,11^ causes short-term air-quality deteriorations. Fireworks lead to elevated concentrations of pollutants such as gaseous pollutants (sulphur dioxide and nitrogen oxides), particulate matter (e.g. PM_10_, PM_2.5_), water-soluble ions and metals^1,2,8–10,12–18^. In the Netherlands, only during New Year’s Eve the general public in the entire country sets off fireworks. PM10 concentrations due to fireworks during New Year’s Eve highly exceed PM10 concentrations observed during the rest of the year. Buijsman and colleagues^19^ described that over the period 1993 to 2012, the average PM_10_-concentration in the first hour after the New Year measured by urban monitoring stations in the Netherlands was approximately 550 μg/m^3^, whereas in the rest of the year, hourly PM_10_ concentrations rarely exceed 100 μg/m^3^ and the yearly averaged background PM_10_ concentration in the Netherlands in 2011 was 24 μg/m^3^.

Short-term exposure to air pollution has been associated with several adverse health effects, such as cardiovascular morbidity^20,21^, respiratory morbidity^20^, hospital admissions^20–22^, cardiovascular mortality^21,23,24^, respiratory mortality^23,24^, and non-accidental mortality^24^. Recent studies have found that air pollution is also associated with health effects other than cardiorespiratory morbidity or mortality, such as dementia^25^, child brain structural alterations and cognitive impairment^26^, and diabetes mortality^27^. Additionally, elderly and infants are most susceptible to mortality from short-term acutely elevated air pollution concentrations^28–30^.

Yet, it is not clear how detrimental instantaneous fireworks emissions are for human health. Few studies have addressed the potential adverse health effects of exposure to fireworks emissions. Hirai *et al*. reported a case of acute eosinophilic pneumonia, following inhalation of smoke from fireworks for three consecutive nights^31^. An inventory of diagnoses made on patients admitted to a hospital in Philadelphia during the week of July 4, revealed two cases of asthma exacerbation, one fatal and one near-fatal, following exposure to elevated PM concentrations from fireworks^32^. Smith *et al*. performed a small-scale study (n = 9) around New Year’s festivities in which they found a decrease in pulmonary function in 2 volunteers with a history of respiratory disease, while the 7 subjects without a history of respiratory disease showed no significant change following exposure to firework emissions^33^.

An increase in emergency room visits following a fireworks episode was described by Bach *et al*.^34^. Furthermore, Beig *et al*.^4^ estimated an increase in mortality and morbidity attributed to population exposure to PM_2.5_ and PM_10_ mass concentrations within areas of 2 kilometers radii from the fireworks displays. Godri *et al*.^9^ described in an *in vitro* study a relationship between the oxidative potential of PM and trace metals associated with fireworks, suggesting a potential negative impact of fireworks emissions on health. In this study, we evaluated the association between hourly PM_10_ concentrations, observed during New Year’s Eve fireworks, and daily mortality in the Netherlands. During the research period setting off fireworks was permitted from 31 December 10 am until 1 January 2 am.

## Results

Peak exposures were found between midnight and 1 am.

### Mortality

Summary statistics of daily mortality, PM_10_ concentrations, and daily temperatures are presented in Table 1.

**Table 1:** Summary statistics of mortality, PM_10_ pollution and temperature 1995–2012.

Variable	Mean	Minimum	Maximum
Non-accidental mortality (day^−1^) 27–30 December	406	361	478
Cardiorespiratory mortality (day^−1^) 27–30 December	193	154	261
Non-accidental mortality (day^−1^) 1–4 January	417	373	494
Cardiorespiratory mortality (day^−1^) 1–4 January	200	165	269
PM_10_ (μg/m^3^) 27–30 December	29	17	51
PM_10_ (μg/m^3^) 1 January 0–1 am	305	185	569
PM_10_ (μg/m^3^) 1 January 0–4 am	167	74	416
PM_10_ (μg/m^3^) 1 January 0–6 am	137	55	343
Average temperature 27 December–4 January (°C)	2.4	−12.6	11.6

**Figure 1:**
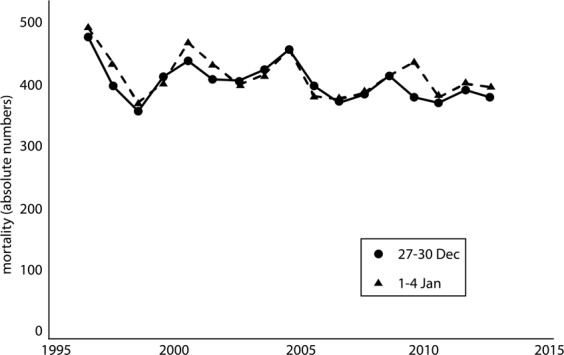
Average daily non-accidental mortality from 27–30 December (1995–2011) and 1–4 January (1996–2012). In the figure the December data are placed in the following year.

Average non-accidental daily mortality decreased from 478 in 1995 to 383 in 2011 (Fig. 1). The decline was mainly found for individuals over 65 years of age (395 in 1995 to 309 in 2011) and for cardiorespiratory mortality (260 in 1995 to 165 in 2011). Daily non-accidental and cardiorespiratory mortality was slightly higher in January as compared to December (Table 1).

### Air quality

**Figure 2:**
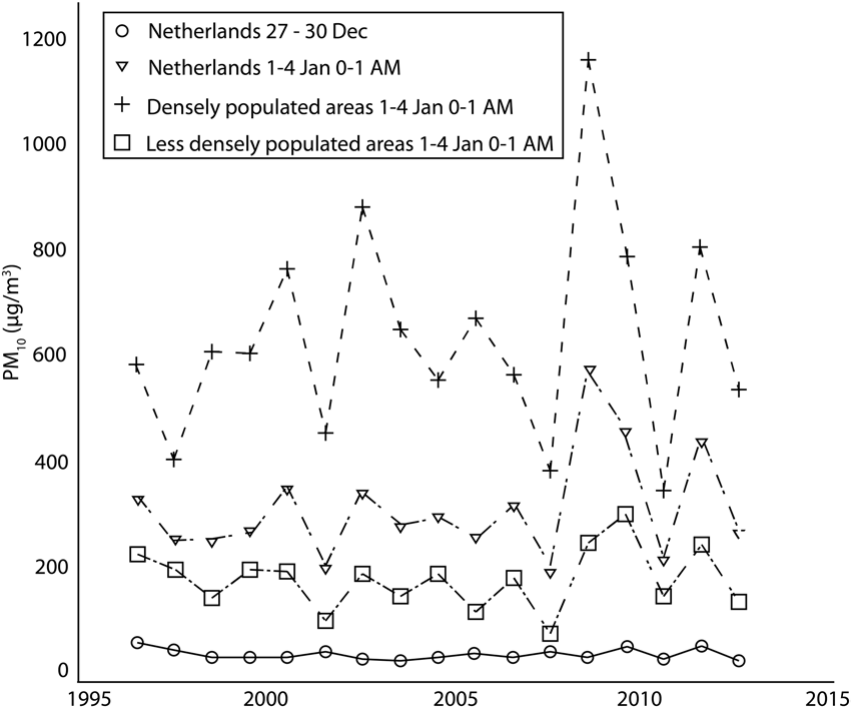
Average hourly PM10 concentrations (μg/m^3^) from 27–30 December (1995–2011) and 1 January 0–1 am (1996–2012). In the figure the December data are placed in the following year.

The average PM_10_ concentration from 27–30 December was 29 μg/m^3^ (Table 1) and slightly decreased (Fig. 2) over the years. PM_10_ concentrations increased during the first hour of the New Year on average in the less densely populated Dutch municipalities by 143 μg/m^3^ (range, 35–255 μg/m^3^), and in the densely populated municipalities by 598 μg/m^3^ (range, 335–1132 μg/m^3^). PM_10_ concentrations during the first hour of 1 January showed no clear pattern over the years (Fig. 2). Figure 2 does not show the PM_10_ concentrations of the ‘densely populated’ and the ‘less densely populated’ municipalities in December separately, because these differences are so small that they would be indiscernible in this figure.

### Associations between daily mortality and PM_10_ from fireworks

Although non-accidental mortality slightly increased following exposure to increased PM_10_ concentrations on exposure days and lag days, none of the associations between the increase in PM_10_ concentrations during the first 6 hours of 1 January and mortality were statistically significant (Table 2). Cardiorespiratory mortality showed some increases and decreases and as with non-accidental mortality none of these associations were statistically significant (Table 2). Results for the increase in PM_10_ concentrations during the first hour (0–1) and the first four hours (0–4) were comparable (Additional file 1: Table S1 and Table S2).

**Table 2:** Mean percent change in daily mortality associated with 10 µg/m^3^ difference in PM_10_ concentration on 1 January 0–6 hours compared to the pre-firework concentration.

	Mean (%)	95% CI
Non-accidental mortality		
1 January	0.075	−0.310, 0.460
2 January	0.188	−0.319, 0.695
3 January	0.094	−0.402, 0.591
4 January	0.073	−0.472, 0.617
1–4 January	0.108	−0.210, 0.426
Cardiorespiratory mortality		
1 January	0.198	−0.410, 0.807
2 January	−0.135	−0.708, 0.438
3 January	−0.101	−0.778, 0.577
4 January	0.168	−0.729, 1.066
1–4 January	0.033	−0.439, 0.504

**Figure 3:**
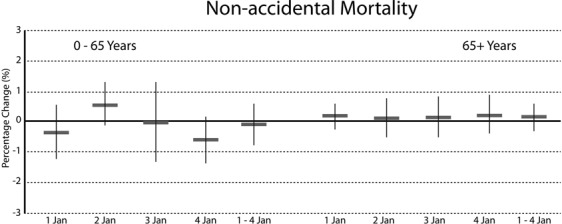
Mean percent increase (95% CI) in daily non-accidental mortality for 0–65 years and over 65 years associated with 10 µg/m^3^ PM10 concentration on 1 January 0–6 hours.

**Figure 4:**
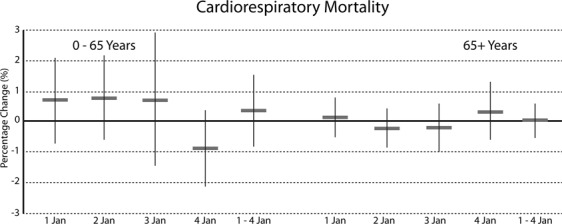
Mean percent increase (95% CI) in daily cardiorespiratory mortality for 0–65 years and over 65 years associated with 10 µg/m^3^ PM10 concentration on 1 January 0–6 hours.

Results for the different age groups are shown in Figs 3 and 4. Results for the densely and the less densely populated regions separately are presented in the Supplemental material (Additional file 1: Table S3 and Table S4). In the densely populated regions no associations between PM_10_ concentrations during the first hours of 1 January and daily non-accidental and cardiorespiratory mortality were found. In the less densely populated areas a 10 μg/m^3^ increase in PM_10_ concentration from 0–6 am 1 January, increased the risk of non-accidental mortality for 0–65 years on 1 January by 1.68% (95%CI: 0.64%, 2.72%). Adjusted for daily temperature the increase was 1.53% (95%CI: 0.36%, 2.70%) as presented in the Supplemental material (Additional file 1: Table S5 and Table S6). No other associations with and without adjustment for daily temperature were found for other age groups.

The case-crossover analysis showed both positive associations between firework-events and mortality, and between PM_10_ concentrations and mortality (Table 3). The same pattern of associations was found amongst the age group over 65 years (Additional file 1: Tables S8 and S9). However, if we put firework-events and PM_10_ levels in the model together, associations strongly decreased (Additional file 1: Table S10).

**Table 3:** Case-crossover analysis in The Netherlands: ORs and 95% CIs for daily mortality associated with fireworks adjusted for temperature and daily mortality associated with 10 µg/m^3^ PM_10_ concentration on hour 0–6 adjusted for daily temperature.

	OR	CI95%
Non-accidental mortality		
**Model: firework-days and temperature**		
1 January (lag 0)	1.003	0.976, 1.030
2 January (lag 1)	1.042	1.015, 1.070**
3 January (lag 2)	1.041	1.015, 1.069**
4 January (lag 3)	1.055	1.028, 1.083***
**Model: PM**_**10**_ **and temperature**		
1 January (lag 0)	1.000	0.998, 1.002
2 January (lag 1)	1.004	1.002, 1.006***
3 January (lag 2)	1.003	1.001, 1.005*
4 January (lag 3)	1.004	1.002, 1.006*
**Cardiorespiratory mortality**		
**Model: firework-days and temperature**		
1 January (lag 0)	1.018	0.980, 1.058
2 January (lag 1)	1.018	0.980, 1.057
3 January (lag 2)	1.055	1.016, 1.095**
4 January (lag 3)	1.072	1.032, 1.113***
**Model: PM**_**10**_ **and temperature**		
1 January (lag 0)	1.001	0.999, 1.004
2 January (lag 1)	1.002	0.999, 1.005
3 January (lag 2)	1.003	1.001, 1.006*
4 January (lag 3)	1.006	1.003, 1.009***

*p < 0.05.

**p < 0.01.

***p < 0.001.

### Sensitivity analyses of the linear regression analysis

No associations between PM_10_ concentrations and mortality were found in the sensitivity analyses for monitoring stations that had data for at least 15 years (results not shown). The results of the sensitivity analyses in which we excluded specific years are given in the supplement (Additional file 1: Table S7). The results of these sensitivity analyses did not show consistent positive associations between PM_10_ and non-accidental and cardiorespiratory mortality.

## Discussion

In general, linear regression analysis revealed no statistically significant associations between PM_10_ concentrations during the first hours of 1 January and daily non-accidental and cardiorespiratory mortality on the first 4 days of January. Additionally, no associations were found in the age group over 65 years. Yet, in a symmetric bi-directional case-crossover analysis we did find positive associations between PM_10_ concentrations and mortality, and between firework-events and mortality using PM_10_ concentrations and firework-events separately. Additionally, the same pattern was found in the age group over 65 years. However, associations considerably weakened when both variables were put in the model together.

Based on the results of the linear regression analysis, effect estimates for non-accidental mortality increase per 10 µg/m^3^ PM_10_ in our study were between 0.1% and 0.2% whereas the non-accidental increases reported earlier in the Netherlands were between 0.3 and 0.8%^24,35^. The increases reported in international meta-analyses were between 0.36% and 0.48%^36,37^. Effect estimates are also lower than the increase (0.49%) associated with PM_10_ from wildfire exposures^23^.

The absence of associations in the linear regression analysis of this study that contrast with the associations found in the abovementioned studies^23,24,35,37^, are potentially due to the short exposure duration to these peak concentrations. Consequently, adverse health effects might be limited, as has been suggested earlier^10^. The peak PM_10_ concentrations lasted for a very short time, found on 1 January from 0–1 hours after which the concentrations quickly leveled off during the following hours as supported by other studies^10,19^. Therefore, daily averaged concentrations PM_10_ on 1 January were lower than the reported 6-h concentrations. Because the results of the analyses with the peak concentrations from 0–1 am and the concentrations of the 6-h time period did not differ, we reported mainly the analyses of the 6-h period to allow for a better comparison to the literature. On the other hand, using the case-crossover approach we did find associations between PM_10_ concentrations and mortality. Because PM_10_ concentrations are strongly associated with New Year’s celebrations, the found associations might also be caused by other putative determinants that are typical for New Year’s celebrations, such as emotional stress, changes in diet and alcohol consumption, and so forth^38^. Therefore, we additionally performed a case-crossover analysis using the Christmas days during our study period as case days. Christmas has many of the abovementioned determinants in common with New Year’s celebrations, with the exception of elevated PM_10_ concentrations. No associations were found between Christmas days and mortality, while we did find an association with New Year’s celebrations. A putative explanation of the difference between Christmas and New Year might be that air pollution due to fireworks caused increased daily mortality.

There are potential alternative explanations for the negative results of the linear regression analysis in our study. First, the data set was limited to 17 periods (i.e. 17 data points) because air pollution by fireworks was restricted to one episode per year (1996–2012). Furthermore, around 75% of the Dutch inhabitants live in the less densely populated municipalities, in which the air quality is relatively less affected by the fireworks than in the densely populated municipalities. However, in the densely populated areas no significant positive associations were found between PM_10_ and daily non-accidental mortality. Therefore, our study may have had insufficient power. Second, susceptible groups such as people with asthma and the elderly probably reduce the exposure to fireworks emissions by staying inside during the peak of firework events^39,40^. Third, the Christmas season, which includes New Year’s Day, is an annually recurring period characterized by days off, eating, drinking, celebrating and stress. The influence of these factors on daily mortality might obscure potentially existing associations with air quality. However, this is not supported by the results from the case-crossover analyses which suggest that factors such as eating, drinking and celebrating might be insufficient to totally obscure the impact of particulate matter on health. The case-crossover design has the advantage of controlling for potential confounders caused by time-invariant individual characteristics (e.g. age, sex, body mass index, and comorbidity)^41–44^. The results of a simulation study by Bateson and Schwartz showed that the symmetric case-crossover design performed best in terms of bias^41^. Unlike other studies, the results in our study obtained with a case-crossover analysis were not similar to those obtained with a different statistical analysis^44,45^. At present, we cannot give insight in what may have caused the difference between the results obtained with the case-crossover analysis and the linear regression analysis, respectively.

A limitation of the study was that we only adjusted for daily temperature. Given the low number of data points we did not adjust for relative humidity and influenza. Furthermore, measurements did not discriminate between fireworks and other sources of particulate matter. However, because of the steep increase of PM_10_ within a short period of time, it is most likely that the peak concentrations on 1 January consisted largely of particulate matter from fireworks. Moreover, there are no indications that other human activities, which emit particulate matter, increase during setting of fireworks^46^.

In this study, PM_10_ was chosen as a proxy for air pollution by fireworks because of the relative abundance of the monitoring data. Although PM_10_ is just one of the pollutants emitted by setting off fireworks, others have shown that the changes in PM_10_ levels correspond with changes in PM_2.5_, PM_1_, carbon monoxide, sulphur dioxide and other pollutants^2–4,13,17,46,47^. Therefore, we think that the use of PM_10_ instead of PM_2.5_ or other pollutants will not have led to exposure misclassification.

To date only a handful of small scale studies have addressed the potential adverse health effects stemming from exposure to firework emissions. In 2016 Lin reviewed evidence related to ambient particulate matter during firework periods and associated human health risks^48^. According to Lin, among the 49 reviewed articles, only 7 have reported health risk evaluations directly related to particulate matter from fireworks. Smith and Dinh described a 26% decrease in maximal midexpiratory flow rate (FEF_25–75%_) following exposure to air pollution from fireworks in two subjects with a history of chronic respiratory disease. No significant decrease was found in the FEF_25–75%_ of seven healthy subjects^33^. Do *et al*. found that post-firework particulate matter was more toxic to human bronchial epithelial BEAS-2B cells than pre-firework particulate matter^49^. The other reviewed studies described quantifications of adverse health impacts caused by exposure to particulate matter^4,50^ or associated metals^16,18,51^. Furthermore, two case studies reported a case of acute eosinophilic pneumonia and a fatal, and a near-fatal asthma exacerbation of two asthmatic children following exposure to elevated PM concentration from fireworks^31,32^. Bach *et al*. described an increase in emergency room visits following a fireworks episode^34^. Godri *et al*.^9^ described in an *in vitro* study a relationship between the oxidative potential of PM and trace metals associated with fireworks, suggesting a potential negative impact of fireworks emissions on health.

## Conclusion

As far as we know, this is the first large-scale observational epidemiological study on the association between exposure to air pollution by firework events and mortality. Although positive associations were absent in the linear regression analysis, the case-crossover analysis showed some positive associations between firework-events, PM_10_ concentrations and daily mortality. As for now, we do not know why the different analysis methods resulted in dissimilar results. Therefore, in light of the contradictory results obtained with the different statistical analyses, we recommend further epidemiological research on the consequences of exposure to firework emissions.

## Methods

### Data collection

#### Mortality

Data on daily non-accidental mortality (excluding external causes, ICD– 10 (V01– Y89) and ICD– 9 (E800– E999) for the total Dutch population for the years 1995–2012 were obtained from Statistics Netherlands^52^. In addition to daily non-accidental mortality we analyzed cause-specific mortality data for cardiorespiratory mortality defined as total cardiovascular mortality, and total respiratory mortality, combined, ICD– 9 (390–459, 460–519) or ICD– 10 (I00– I99, J00– J99).

Mortality data were stratified in age groups 0–65 years and older than 65 years. Furthermore, data were stratified according to population density (‘densely populated’: municipalities with ≥2500 inhabitants/km^2^ and ‘less densely populated’: all other municipalities in the Netherlands).

#### Air quality

PM_10_ data were obtained from the National Institute for Public Health and the Environment (RIVM), which operates the Ambient Air Quality Monitoring Network in the Netherlands and used as a proxy for air pollution by fireworks. The network had 46 monitoring stations for PM_10_ in both densely and less densely populated areas during the period 1995–2012^53^.

For the entire period, concentration differences between 1-h average concentrations on 1 January and the mean of 1-h average concentrations over 27–30 December of the preceding year were calculated for each monitoring station. Besides the 1-h average concentrations from 0–1 am, the 4-hours averaged concentrations from 0–4 am and the 6-hours averaged concentrations from 0–6 am on 1 January were used.

Imputation of one or more missing values between 1 am and 6 am on 1 January was based on linear interpolation of preceding and following 1-h average concentrations per monitoring station. Missing values between midnight and 1 am were not imputed, because maximum levels were found from 0–1 am.

#### Meteorology

Mean daily temperature was obtained from the Royal Dutch Meteorological Institute (KNMI) for a weather station at a central rural location (de Bilt, 05° 17′ 70″, 52° 10′ 10″) of the national meteorological network^54^.

### Data analysis

#### Linear regression analysis

SPSS version 20.0 statistical software was used (SPSS Inc., Chicago, IL, USA). Associations between exposure variables and mortality with and without adjustment for daily temperature were calculated using a linear regression analysis. The level of statistical significance was set at *p* < 0.05.

Daily mortality differences between 1 January, 2 January, 3 January, 4 January, the average over 1–4 January and the average daily mortality over 27–30 December of the preceding year were analyzed in association with PM_10_ concentration differences (per 10 µg/m^3^) between the 1-h average concentrations during which the peak concentration occurs on 1 January (0–1 am and 0–6 am) and the 1-h average concentrations over 27–30 December of the preceding year. Regression coefficients were recalculated to mean percent increase (95% CI) in daily mortality per 10 µg/m^3^ PM10 concentration on 1 January 0–6 hours. Therefore, data were restricted to 17 periods of four days before (27–30 December) and four days after (1–4 January) New Year. Stratified data for different age groups and population densities were analyzed separately. For the analyses stratified by population densities we used PM_10_ data for stations in densely populated and less densely populated areas separately. For each analysis, we calculated the percent change in mortality per 10 µg/m^3^ increase in PM_10_ by dividing the regression coefficient for PM_10_ by the baseline daily mortality (i.e. the age-group-specific and cause-specific average daily mortality on 27–30 December during the entire study period).

#### Case-crossover analysis

Additionally, a case-crossover analysis^55,56^ was used in which each deceased individual served as her or his own control. We used the symmetric bi-directional approach^41^, in which the 1 January was selected as case day (see first column) and 4 control days were chosen per case day (the same days of the week in the 2 preceding weeks and in the 2 successive weeks). PM_10_ concentrations were calculated as the mean of the first six hours per day. Using conditional logistic regression, we estimated the OR in three models for mortality associated with 1) firework-days adjusted for temperature, 2) 10 µg/m^3^ PM_10_ concentration adjusted for temperature, and 3) 10 µg/m^3^ PM_10_ concentration adjusted for temperature and firework-day (yes/no). For both the case day and PM_10_ concentrations four lags were evaluated (lags 0, 1, 2, and 3 days).

#### Sensitivity analysis of the linear regression analysis

Sensitivity analyses were performed by analyzing only monitoring stations that had data available during the entire measurement period (n = 8), and by analyzing stations that had data available for at least 15 of the 17 years (n = 16). We also performed sensitivity analyses to investigate if the results changed when we excluded: a. the end-of-year- period with the highest level of PM_10_; b. the end-of-year-period with the lowest level of PM_10_ and c. the two end-of-year-periods with the highest level of PM_10_ (there were 2 periods with much higher levels than the other years (see Fig. 2)).

## Supplementary information

SI Air pollution during New Years fireworks and mortality

## Data Availability

The datasets generated during and/or analyzed during the current study are available from the corresponding author on reasonable request.
