# Supplementary material for: Air pollution during New Year’s fireworks and daily mortality in the Netherlands
Source: Sci Rep. 2019 Apr 5;9:5735. doi: 10.1038/s41598-019-42080-6 (PMC6450968; doi:10.1038/s41598-019-42080-6)
Supplement: Supplementary file 1 — SI Air pollution during New Years fireworks and mortality [file 41598_2019_42080_MOESM1_ESM.doc]

## Journal: Scientific Reports

## Title:

## Air pollution during New Year’s fireworks and daily mortality in the Netherlands

Frans E. Greven^1^, Judith M. Vonk^2,3^, Paul Fischer^4^, Frans Duijm^1^, Nienke M. Vink^1^, Bert Brunekreef^5,6^

1. Department of environmental health, municipal health services Groningen, PO Box 584, 9700 AN, Groningen, the Netherlands.

Corresponding author: frans.greven@ggd.groningen.nl

2. Department of epidemiology, university of Groningen, university medical center Groningen, PO Box 30.001, 9700 RB, Groningen, the Netherlands.

3. Groningen research institute on asthma and COPD (GRIAC), university of Groningen, university medical center Groningen, PO Box 30.001, 9700 RB, Groningen, the Netherlands.

4. Centre for Sustainability, Environment and Health; Department for Environmental Health, National Institute of Public Health and the Environment, RIVM, PO Box 1, 3720 BA, Bilthoven, the Netherlands.

5. Institute for Risk Assessment Sciences, University of Utrecht, Yalelaan 2, 3584 CM, Utrecht, the Netherlands.

6. Julius Center for Health Sciences and Primary Care, University Medical Center Utrecht, 3584 CJ, Utrecht, the Netherlands.

## Air pollution during New Year’s fireworks and daily mortality in the Netherlands

# Online Supplement

Table S1. Mean percent change in daily mortality associated with 10 µg/m^3^ PM10 concentration on 1 January 0-1 hours.

|  | |  |  | |
| --- | --- | --- | --- | --- |
|  | Mean (%) | | | 95% CI |
| Non-accidental mortality | | | | |
| 1 January | -0.026 | | | -0.316, 0.264 |
| 2 January | 0.144 | | | -0.235, 0.524 |
| 3 January | 0.051 | | | -0.322, 0.425 |
| 4 January | 0.043 | | | -0.366, 0.451 |
| 1-4 January | 0.053 | | | -0.188, 0.294 |
| Cardiorespiratory mortality | | | | |
| 1 January | -0.004 | | | -0.468, 0.459 |
| 2 January | -0.072 | | | -0.503, 0.359 |
| 3 January | -0.032 | | | -0.567, 0.477 |
| 4 January | 0.070 | | | -0.604, 0.745 |
| 1-4 January | -0.009 | | | -0.363, 0.344 |

Table S2. Mean percent change in daily mortality associated with 10 µg/m^3^ PM10 concentration on 1 January 0-4 hours.

|  | |  |  | |
| --- | --- | --- | --- | --- |
|  | Mean (%) | | | 95% CI |
| Non-accidental mortality | | | | |
| 1 January | 0.054 | | | -0.278, 0.385 |
| 2 January | 0.165 | | | -0.270, 0.601 |
| 3 January | 0.069 | | | -0.359, 0.496 |
| 4 January | 0.061 | | | -0.407, 0.529 |
| 1-4 January | 0.087 | | | -0.187, 0.361 |
| Cardiorespiratory mortality | | | | |
| 1 January | 0.160 | | | -0.364, 0.684 |
| 2 January | -0.099 | | | -0.593, 0.395 |
| 3 January | -0.076 | | | -0.659, 0.507 |
| 4 January | 0.132 | | | -0.640, 0.904 |
| 1-4January | 0.029 | | | -0.376, 0.435 |

Table S3. Mean percent change in daily mortality in the densely populated municipalities associated with 10 µg/m^3^ PM10 concentration on 1 January 0-6 hours.

|  |  |  |  |  |  |  |
| --- | --- | --- | --- | --- | --- | --- |
| Non-accidental mortality | | | | | | |
|  | All ages | | 0-65 year | | 65+ year | |
|  | Mean (%) | 95% CI | Mean (%) | 95% CI | Mean (%) | 95% CI |
| 1 January | 0.088 | -0.367, 0.543 | 0.061 | -0.910,1.033 | 0.094 | -0.432, 0.619 |
| 2 January | -0.053 | -0.504, 0.397 | -0.411 | -1.564, 0.742 | 0.021 | -0.455, 0.498 |
| 3 January | 0.207 | -0.201, 0.614 | -0.493 | -1.635, 0.649 | 0.353 | -0.115, 0.821 |
| 4 January | -0.269 | -0.783, 0.245 | -0.628 | -1.495, 0.239 | -0.194 | -0.766, 0.377 |
| 1-4 January | -0.007 | -0.363, 0.349 | -0.368 | -1.102, 0.366 | 0.068 | -0.317, 0.454 |
| Cardiorespiratory mortality | | | | | | |
|  | All ages | | 0-65 year | | 65+ year | |
|  | Mean (%) | 95% CI | Mean (%) | 95% CI | Mean (%) | 95% CI |
| 1 January | 0.020 | -0.743, 0.783 | 0.944 | -1.451, 3.340 | -0.089 | -0.822, 0.644 |
| 2 January | -0.109 | -0.566, 0.349 | -0.169 | -2.638, 2.298 | -0.101 | -0.606, 0.403 |
| 3 January | 0.279 | -0.407, 0.966 | -0.839 | -2.833, 1.155 | 0.411 | -0.331, 1.154 |
| 4 January | -0.370 | -1.139, 0.399 | -1.324 | -2.883, 0.235 | -0.258 | -1.052, 0.536 |
| 1-4 January | -0.045 | -0.558, 0.468 | -0.346 | -1.668, 0.974 | -0.009 | -0.545, 0.527 |

Table S4. Mean percent change in daily mortality in the less densely populated municipalities associated with 10 µg/m^3^ PM10 concentration on 1 January 0-6 hours.

|  |  |  |  |  |  |  |
| --- | --- | --- | --- | --- | --- | --- |
| Non-accidental mortality | | | | | | |
|  | All ages | | 0-65 year | | 65+ year | |
|  | Mean (%) | 95% CI | Mean (%) | 95% CI | Mean (%) | 95% CI |
| 1 January | 0.195 | -0.513, 0.903 | -0.395 | -2.219, 1.428 | 0.327 | -0.463, 1.117 |
| 2 January | 0.412 | -0.489, 1.313 | 1.679 | 0.641, 2.716 | 0.129 | -0.945, 1.203 |
| 3 January | 0.232 | -0.745, 1.210 | 0.844 | -1.603, 3.291 | 0.096 | -1.008, 1.200 |
| 4 January | 0.520 | -0.413, 1.453 | -0.464 | -1.896, 0.968 | 0.739 | -0.336, 1.815 |
| 1-4 January | 0.340 | -0.265, 0.945 | 0.416 | -0.831, 1.663 | 0.323 | -0.438, 1.084 |
| Cardiorespiratory mortality | | | | | | |
|  | All ages | | 0-65 year | | 65+ year | |
|  | Mean (%) | 95% CI | Mean (%) | 95% CI | Mean (%) | 95% CI |
| 1 January | 0.336 | -0.988, 1.659 | 0.963 | -1.454, 3.380 | 0.252 | -1.201, 1.705 |
| 2 January | -0.396 | -1.467, 0.674 | 1.427 | -0.458, 3.312 | -0.639 | -1.803, 0.524 |
| 3 January | -0.252 | -1.469, 0.965 | 3.095 | -0.653, 6.844 | -0.699 | -2.141, 0.744 |
| 4 January | 0.900 | -0.658, 2.459 | -0.416 | -3.175, 2.344 | 1.076 | -0.620, 2.771 |
| 1-4 January | 0.147 | -0.678, 0.971 | 1.267 | -0.734, 3.269 | -0.003 | -0.973, 0.968 |

Table S5. Mean percent change in daily mortality in the densely populated municipalities associated with 10 µg/m^3^ PM10 concentration on 1 January 0-6 hours adjusted for daily temperature.

|  |  |  |  |  |  |  |
| --- | --- | --- | --- | --- | --- | --- |
| Non-accidental mortality | | | | | | |
|  | All ages | | 0-65 year | | 65+ year | |
|  | Mean (%) | 95% CI | Mean (%) | 95% CI | Mean (%) | 95% CI |
| 1 January | 0.125 | -0.279, 0.528 | 0.135 | -0.748, 1.016 | 0.123 | -0.390, 0.635 |
| 2 January | -0.049 | -0.522, 0.424 | -0.424 | -1.634, 0.785 | 0.030 | -0.469, 0.529 |
| 3 January | 0.187 | -0.230, 0.604 | -0.516 | -1.709, 0.677 | 0.332 | -0.149, 0.816 |
| 4 January | -0.263 | -0.791, 0.265 | -0.610 | -1.480, 0.260 | -0.190 | -0.783, 0.402 |
| 1-4 January | 0.002 | -0.077, 0.126 | -0.353 | -1.117, 0.411 | 0.077 | -0.325, 0.478 |
| Cardiorespiratory mortality | | | | | | |
|  | All ages | | 0-65 year | | 65+ year | |
|  | Mean (%) | 95% CI | Mean (%) | 95% CI | Mean (%) | 95% CI |
| 1 January | 0.064 | -0.676, 0.803 | 1.014 | -1.445, 3.472 | -0,048 | -0.762, 0.665 |
| 2 January | -0.141 | -0.600, 0.317 | -0.157 | -2.750, 2.438 | -0,140 | -0.643, 0.363 |
| 3 January | 0.228 | -0.451, 0.906 | -0.940 | -2.978, 11.037 | 0,366 | -0.383, 1.114 |
| 4 January | -0.375 | -1.174, 0.425 | -1.336 | -2.953, 0.280 | -0,261 | -1.087, 0.565 |
| 1-4 January | -0.053 | -0.588, 0.481 | -0.352 | -1.734, 0.370 | -0,018 | -0.178, 0.130 |

Table S6. Mean percent change in daily mortality in the less densely populated municipalities associated with 10 µg/m^3^ PM10 concentration on 1 January 0-6 hours adjusted for daily temperature.

|  |  |  |  |  |  |  |
| --- | --- | --- | --- | --- | --- | --- |
| Non-accidental mortality | | | | | | |
|  | All ages | | 0-65 year | | 65+ year | |
|  | Mean (%) | 95% CI | Mean (%) | 95% CI | Mean (%) | 95% CI |
| 1 January | 0.228 | -0.560, 1.016 | -0.435 | -2.468, 1.598 | 0.376 | -0.502, 1.254 |
| 2 January | 0.382 | -0.647, 1.412 | 1.529 | 0.361, 2.698 | 0.127 | -1.101, 1.355 |
| 3 January | -0.050 | -1.069, 0.969 | 0.639 | -2.104, 3.382 | -0.203 | -1.365, 0.959 |
| 4 January | 0.466 | -0.533, 1.465 | -0.576 | -2.100, 0.948 | 0.698 | -0.458, 1.854 |
| 1-4 January | 0.189 | -0.454, 0.832 | 0.257 | -1.128, 1.643 | 0.174 | -0.654, 1.002 |
| Cardiorespiratory mortality | | | | | | |
|  | All ages | | 0-65 year | | 65+ year | |
|  | Mean (%) | 95% CI | Mean (%) | 95% CI | Mean (%) | 95% CI |
| 1 January | 0.336 | -1.140, 1.812 | 1.242 | -1.416, 3.900 | 0.215 | -1.404, 1.834 |
| 2 January | -0.450 | -1.672, 0.773 | 1.974 | -0.044, 3.992 | -0.773 | -2.091, 0.545 |
| 3 January | -0.697 | -1.899, 0.505 | 2.902 | -1.316, 7.120 | -1.177 | -2.639, 0.286 |
| 4 January | 0.798 | -0.867, 2.464 | -0.405 | -3.380, 2.571 | 0.959 | -0.851, 2.769 |
| 1-4 January | -0.101 | -0.953, 0.750 | 1.429 | -0.814, 3.672 | -0.305 | -1.301, 0.690 |

Table S7. Mean percent change in daily mortality associated with 10 µg/m^3^ PM10 concentration on 1 January 0-6 hours adjusted for daily temperature in the Netherlands in which a. the end-of-year- period with the highest level of PM10, b. the end-of-year-period with the lowest level of PM10, and c. the two end-of-year-periods with the highest level of PM10 were excluded.

|  |  |  |  |  |  |  |
| --- | --- | --- | --- | --- | --- | --- |
| Non-accidental mortality – all ages | | | | | | |
|  | a. | | b. | | c. | |
|  | Mean (%) | 95% CI | Mean (%) | 95% CI | Mean (%) | 95% CI |
| 1 January | 0.320 | -0.269, 0.416 | 0.166 | -0.253, 0.586 | -0.364 | -0.937, 0.801 |
| 2 January | 0.554 | -0.233, 1.340 | 0.305 | -0.202, 0.811 | -0.068 | -1.200, 1.051 |
| 3 January | 0.380 | -0.300, 1.060 | -0.045 | -0.560, 0.469 | -0.074 | -0.923, 1.132 |
| 4 January | 0.253 | -0.631, 1.136 | 0.120 | -0.491, 0.732 | 0.104 | -1.680, 1.025 |
| 1-4 January | 0.347 | -0.117, 0.810 | 0.118 | -0.222, 0.459 | -0.328 | -0.745, 0.471 |
| Cardiorespiratory mortality – all ages | | | | | | |
|  | a. | | b. | | c. | |
|  | Mean (%) | 95% CI | Mean (%) | 95% CI | Mean (%) | 95% CI |
| 1 January | -0.000 | -0.970, 0.970 | 0.380 | -0.261, 1.022 | -0.816 | -2.178, 0.546 |
| 2 January | -0.284 | -1.224, 0.655 | 0.039 | -0.389, 0.468 | -0.838 | -2.245, 0.568 |
| 3 January | -0.111 | -1.063, 0.841 | -0.239 | -0.898, 0.420 | -0.641 | -2.042, 0.760 |
| 4 January | 0.351 | -1.087, 1.790 | 0.303 | -0.627, 1.235 | -0.477 | -2.707, 1.752 |
| 1-4 January | -0.058 | -0.127, 0.026 | 0.090 | -0.314, 0.495 | -0.761 | -1.749, 0.227 |

Table S8. Case-crossover analysis in The Netherlands: ORs and 95% CIs for daily mortality associated firework-day (yes/no) adjusted for daily temperature.

|  |  |  |  |  |  |  |  | |  |  |
| --- | --- | --- | --- | --- | --- | --- | --- | --- | --- | --- |
| Non-accidental mortality | | | | | | | | | | |
|  | All ages | | | 0-65 year | | | | 65+ year | | |
|  | OR | 95% CI | p | OR | 95% CI | p | OR | | 95% CI | p |
| 1 January | 1.003 | 0.976, 1.030 | 0.840 | 0.985 | 0.925, 1.049 | 0.635 | 1.007 | | 0.978, 1.037 | 0.656 |
| 2 January | 1.042 | 1.015, 1.070* | 0.002 | 1.049 | 0.986, 1.116 | 0.127 | 1.041 | | 1.011, 1.071* | 0.007 |
| 3 January | 1.041 | 1.015, 1.069 | 0.002 | 1.030 | 0.969, 1.095 | 0.346 | 1.044 | | 1.014, 1.074* | 0.003 |
| 4 January | 1.002 | 0.999, 1.005 | 0.243 | 1.031 | 0.969, 1.096 | 0.399 | 1.061 | | 1.031, 1.092* | 0.000 |
| Cardiorespiratory mortality | | | | | | | | | | |
|  | All ages | | | 0-65 year | | | | 65+ year | | |
|  | OR | 95% CI | p | OR | 95% CI | p | OR | | 95% CI | p |
| 1 January | 1.018 | 0.980, 1.058 | 0.362 | 1.055 | 0.945, 1.178 | 0.340 | 1.013 | | 0.972, 1.055 | 0.536 |
| 2 January | 1.018 | 0.980, 1.057 | 0.358 | 1.051 | 0.941, 1.174 | 0.376 | 1.014 | | 0.973, 1.056 | 0.510 |
| 3 January | 1.055 | 1.016, 1.095* | 0.005 | 1.025 | 0.919, 1.144 | 0.654 | 1.059 | | 1.018, 1.102* | 0.005 |
| 4 January | 1.072 | 1.032, 1.113* | 0.000 | 1.015 | 0.906, 1.137 | 0.802 | 1.080 | | 1.037, 1.124* | 0.000 |

Table S9. Case-crossover analysis in The Netherlands: ORs and 95% CIs for daily mortality associated with 10 µg/m3 PM10 concentration on hour 0-6 adjusted for daily temperature.

|  |  |  |  |  |  |  |  |  |  |
| --- | --- | --- | --- | --- | --- | --- | --- | --- | --- |
| Non-accidental mortality | | | | | | | | | |
|  | All ages | | | 0-65 year | | | 65+ year | | |
|  | OR | 95% CI | p | OR | 95% CI | p | OR | 95% CI | p |
| 1 January | 1.000 | 0.998, 1.002 | 0.941 | 0.997 | 0.992, 1.002 | 0.179 | 1.001 | 0.998, 1.003 | 0.582 |
| 2 January | 1.004 | 1.002, 1.006* | 0.000 | 1.006 | 1.001, 1.010* | 0.016 | 1.003 | 1.001, 1.005* | 0.003 |
| 3 January | 1.003 | 1.001, 1.005* | 0.002 | 1.002 | 0.997, 1.006 | 0.455 | 1.003 | 1.001, 1.006* | 0.002 |
| 4 January | 1.004 | 1.002, 1.006* | 0.000 | 1.002 | 0.997, 1.007 | 0.386 | 1.005 | 1.003, 1.007* | 0.000 |
| Cardiorespiratory mortality | | | | | | | | | |
|  | All ages | | | 0-65 year | | | 65+ year | | |
|  | OR | 95% CI | p | OR | 95% CI | p | OR | 95% CI | p |
| 1 January | 1.001 | 0.999, 1.004 | 0.338 | 1.005 | 0.997, 1.014 | 0.241 | 1.001 | 0.998, 1.004 | 0.545 |
| 2 January | 1.002 | 0.999, 1.005 | 0.123 | 1.007 | 0.998, 1.015 | 0.122 | 1.002 | 0.999, 1.005 | 0.280 |
| 3 January | 1.003 | 1.001, 1.006* | 0.020 | 1.004 | 0.995, 1.012 | 0.388 | 1.003 | 1.000, 1.006* | 0.030 |
| 4 January | 1.006 | 1.003, 1.009* | 0.000 | 1.001 | 0.992, 1.010 | 0.814 | 1.006 | 1.003, 1.009* | 0.000 |

Table S10. Case-crossover analysis in The Netherlands: ORs and 95% CIs for daily mortality associated with 10 µg/m3 PM10 concentration on hour 0-6 adjusted for daily temperature and firework-day (yes/no).

|  |  |  |  |  |  |  |  |  |  |
| --- | --- | --- | --- | --- | --- | --- | --- | --- | --- |
| Non-accidental mortality | | | | | | | | | |
|  | All ages | | | 0-65 year | | | 65+ year | | |
|  | OR | 95% CI | p | OR | 95% CI | p | OR | 95% CI | p |
| 1 January | 0.999 | 0.996, 1.003 | 0.684 | 0.993 | 0.985, 1.001 | 0.099 | 1.001 | 0.997, 1.004 | 0.748 |
| 2 January | 1.003 | 1.000, 1.007 | 0.052 | 1.008 | 1.000, 1.016 | 0.049 | 1.002 | 0.999, 1.006 | 0.222 |
| 3 January | 1.002 | 0.998, 1.005 | 0.318 | 1.000 | 0.992, 1.008 | 0.977 | 1.002 | 0.998, 1.006 | 0.265 |
| 4 January | 1.003 | 0.999, 1.006 | 0.123 | 1.001 | 0.993, 1.009 | 0.874 | 1.003 | 0.999, 1.007 | 0.106 |
| Cardiorespiratory mortality | | | | | | | | | |
|  | All ages | | | 0-65 year | | | 65+ year | | |
|  | OR | 95% CI | p | OR | 95% CI | p | OR | 95% CI | p |
| 1 January | 1.001 | 0.996, 1.006 | 0.711 | 1.005 | 0.990, 1.020 | 0.501 | 1.000 | 0.995, 1.006 | 0.862 |
| 2 January | 1.003 | 0.998, 1.009 | 0.174 | 1.010 | 0.996, 1.025 | 0.157 | 1.003 | 0.997, 1.008 | 0.350 |
| 3 January | 1.000 | 0.995, 1.005 | 0.924 | 1.006 | 0.992, 1.021 | 0.393 | 0.999 | 0.994, 1.005 | 0.835 |
| 4 January | 1.004 | 0.999, 1.009 | 0.087 | 1.000 | 0.985, 1.016 | 0.956 | 1.005 | 0.999, 1.010 | 0.077 |
